# Use of prototype two-channel endoscope with elevator enables larger lift-and-snare endoscopic mucosal resection in a porcine model

**DOI:** 10.1093/gastro/got035

**Published:** 2014-01-07

**Authors:** Matthew Atkinson, Chike Chukwumah, Jeffrey Marks, Amitabh Chak

**Affiliations:** Case Western Reserve University College of Medicine, Cincinnati, Ohio, USA

**Keywords:** endoscopic mucosal resection, endoscopic polypectomy, gastric cancer

## Abstract

**Background:** Flat and depressed lesions are becoming increasingly recognized in the esophagus, stomach, and colon. Various techniques have been described for endoscopic mucosal resection (EMR) of these lesions.

**Aims:** To evaluate the efficacy of lift-grasp-cut EMR using a prototype dual-channel forward-viewing endoscope with an instrument elevator in one accessory channel (dual-channel elevator scope) as compared to standard dual-channel endoscopes.

**Methods:** EMR was performed using a lift-grasp-cut technique on normal flat rectosigmoid or gastric mucosa in live porcine models after submucosal injection of 4 mL of saline using a dual-channel elevator scope or a standard dual-channel endoscope. With the dual-channel elevator scope, the elevator was used to attain further lifting of the mucosa. The primary endpoint was size of the EMR specimen and the secondary endpoint was number of complications.

**Results:** Twelve experiments were performed (six gastric and six colonic). Mean specimen diameter was 2.27 cm with the dual-channel elevator scope and 1.34 cm with the dual-channel endoscope (*P = *0.018). Two colonic perforations occurred with the dual-channel endoscope, vs no complications with the dual-channel elevator scope.

**Conclusions:** The increased lift of the mucosal epithelium, through use of the dual-channel elevator scope, allows for larger EMR when using a lift-grasp-cut technique. Noting the thin nature of the porcine colonic wall, use of the elevator may also make this technique safer.

## INTRODUCTION

As use of endoscopy has increased, more mucosal lesions are discovered with advanced dysplasia or early cancer. There is increasing evidence that endoscopic mucosal resection (EMR) may provide adequate therapy for these lesions. In Japan, early gastric cancer is treated with EMR if the cancer is well differentiated, is <2 cm in size (<1 cm in size if the lesion is depressed), is not associated with an ulcer, and is limited to the mucosa [1]. Similar criteria have been used with success for EMR of high-grade dysplasia of the esophagus [2] and flat or depressed colon adenomas [3–6].

Several techniques of EMR exist. The strip biopsy technique has been successfully applied to early esophageal cancer, in which the cancer is grasped with a snare and resected with electrocautery (similar to a basic polypectomy) [7]. Submucosal injection of saline or other substance is frequently used prior to snare resection (the lift and cut technique), in an attempt to separate the submucosa from the muscularis and decrease risk of perforation [3–6]. In the EMR-cap technique, a snare is opened within the inner perimeter of a cap (similar to that used in band ligation) that was used to move the lesion away from the muscularis by suction, prior to grasping the base with the snare and resecting [8, 9]. The EMR-with-ligation technique actually captured the mucosa with a rubber band (as in variceal ligation) and the banded tissue was then removed with snare cautery [10, 11]. While submucosal injection is used prior to the EMR-cap technique, it may not be necessary with the EMR-with-ligation technique.

One shortcoming of EMR is the inability to perform *en bloc* resection of larger lesions. Endoscopic submucosal dissection is a technique that has been shown to provide better *en bloc* resection rate [12, 13]. However, the technique involves a steep learning curve and is associated with increased rates of bleeding and perforation. It is not generally performed outside specialized centers in Japan. An easier technique for endoscopic mucosal resection of larger lesions is needed.

To address this, two-channel EMR has been evaluated. It was described in porcine models and achieved complete *en bloc* resection of an area of 3 cm of normal gastric mucosa in 9 out of 10 cases in a mean time of 32.4 minutes [14]. The technique was used in 17 patients with flat gastrointestinal polyps and 14 (82%) had complete endoscopic resection with two complications (bleeding in one and perforation in another) [15]. For rectal neuroendocrine tumors <16 mm, the grasp-and-snare technique was shown to be significantly quicker to perform, to have fewer complications, and to provide complete resection in 86.3% of lesions, which was not inferior to endoscopic submucosal dissection [16].

The dual-channel elevator scope (XGIF-2TQ160R3, Olympus Medical Systems Corp.) is a prototype, forward-viewing, dual-channel endoscope with an elevator in one accessory channel. It has been seen to allow faster endoscopic submucosal dissection in the greater and lesser curvature of the *ex vivo* stomach [17]. We propose that EMR using a lift-grasp-elevate-cut technique will allow for larger resection specimens, with fewer complications compared with a lift-grasp-cut technique.

## METHODS

### Study design

We performed a prospective, comparative trial of EMR of normal flat colonic and gastric mucosa in live porcine models with the dual-channel elevator scope vs the standard dual-channel endoscope. Each experiment consisted of one EMR using the dual-channel elevator scope and one EMR using the standard dual-channel endoscope. The order of scopes was randomized by tossing a coin. Resection specimens were collected with the Roth net, spread to maximal diameter by the endoscopist, and maximal diameter was measured with a rule. The polypectomy site was examined endoscopically for evidence of perforation. Following the EMR experiments, the animals were sacrificed and the organs dissected and re-examined for perforation.

### Procedural technique

**Figure 1.**
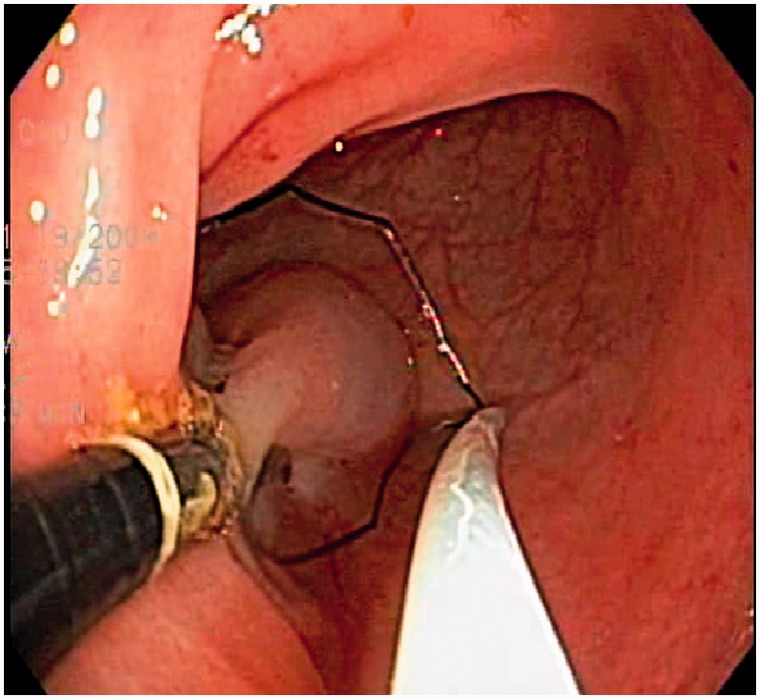
Grasping and lifting the mucosa with the dual-channel endoscope.

**Figure 2.**
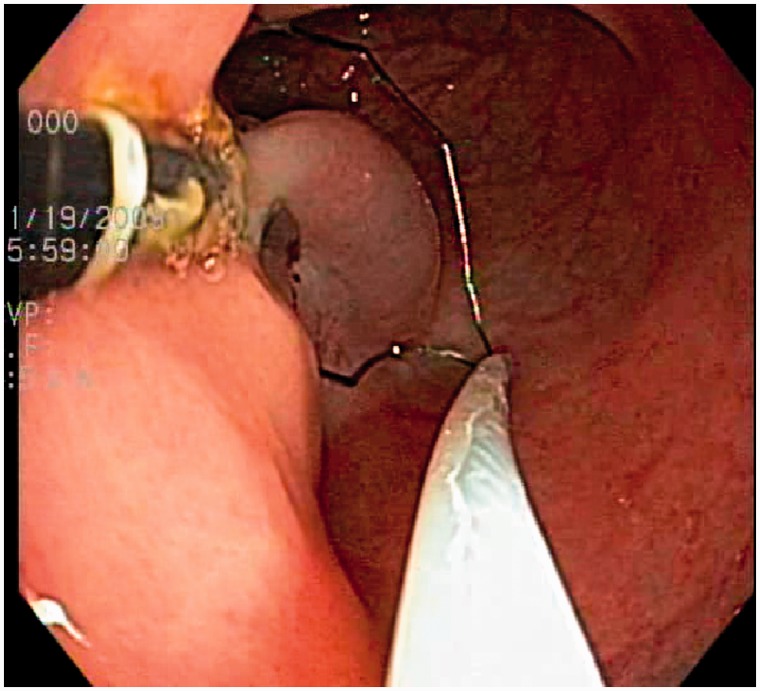
Further lifting of the mucosa in a perpendicular fashion away from the muscularis with the elevator.

A normal flat area of rectosigmoid or gastric mucosa was chosen by the endoscopist. A standard dose of 4 mL of saline was injected submucosally, to lift the mucosa. For the dual-channel upper endoscope, a lift-grasp-cut technique was employed in the following manner: a 15 mm snare (SD-240U-15, Olympus Medical Systems Corp.) was passed down one channel and the rat-tooth forceps (FG-9 L-1, Olympus Medical Systems Corp.) down the other channel. The forceps were passed through the open snare. The saline-lifted mucosa was grasped with the forceps and pulled back until taut (refer to Figure 1). The open snare was then passed over the forceps to grasp the largest possible mucosal segment. Snare cautery was then performed using standard electrosurgical settings. An identical technique was used for the dual-channel elevator scope. However, prior to closing the snare, the mucosa was lifted further by elevating the forceps with the elevator (Figure 2: lift-grasp-elevate-cut technique).

### Statistics

A two-tailed *t*-test was used to compare polypectomy sizes.

## RESULTS

Twelve experiments were performed (six gastric and six colonic). Mean polypectomy size was 2.27 cm (range: 0.5–4.0 cm; standard deviation: 1.15) with the dual-channel elevator scope and 1.34 cm (range: 0.4–2.5 cm; standard deviation: 0.52) with the dual-channel endoscope (*P = *0.018). Two colonic perforations occurred with the dual-channel endoscope, vs none with the dual-channel elevator scope.

## DISCUSSION

EMR with standard suction techniques is limited in the size of specimen that can be removed *en bloc*. Endoscopic submucosal dissection has been used to increase the size of *en bloc* specimen resection. However, this technique takes considerable skill and experience and is primarily used in Japan, where the prevalence of early gastric cancer is high. In this pilot study, we evaluated use of a lift-grasp-elevate-cut technique with a prototype dual-channel upper endoscope with an elevator in one accessory channel. We found that mean EMR diameter was 2.27 cm, significantly larger than had been attained with the standard dual-channel endoscope. Additionally, despite the thin nature of the porcine colon wall, we caused no perforations with the dual-channel elevator scope, compared with two perforations using the standard dual-channel endoscope.

The increased size and safety of EMR specimens probably result from increased lifting of the mucosa and submucosa away from the muscularis in a perpendicular, rather than the tangential, direction allowed by the standard dual-channel endoscope (see Figures 1 and 2). This is similar to the perpendicular lift achieved by the suction EMR techniques. However, the dual-channel elevator scope EMR specimen is not limited to the mucosa suctioned into the cap so, theoretically, broader specimens can be obtained.

The EMR specimen diameter attained with the dual-channel elevator scope of 2.27 cm is similar to other EMR studies, which have a range of diameters from 1.97–2.76 cm [17–21]. However, of the 12 EMR specimens performed with the dual-channel elevator scope, one was 3.5 cm in diameter and two were 4 cm in diameter. Thus, with more experience, it may be possible to consistently achieve larger specimens. Further experience with this technique may broaden the indication for EMR, beyond the typical cut-off of 2 cm. This may permit EMR of lesions that have typically required endoscopic subumucosal resection.

The present study has several limitations. First, it was a feasibility study on a normal mucosa in a porcine model. It is unclear how this would translate to diseased mucosae in humans. Second, the most common EMR techniques currently in use are the suction techniques. Because of the cost of suction EMR kits, direct comparison between the dual-channel elevator scope and suction EMR was not performed. Third, EMR was performed in the area of the stomach or colon that was most comfortable for the endoscopist. It is unclear how the dual-channel elevator scope will perform in more difficult locations (e.g. high on the lesser curve or more proximally in the colon).

In summary, performance of lift-grasp-elevate-cut EMR with the dual-channel elevator scope produces larger specimens with fewer complications than lift-grasp-cut EMR with a standard dual-channel endoscope. Specimens as large as 4 cm were obtained without complications with the dual-channel elevator scope. Direct comparison with suction EMR techniques in the porcine model and consideration of use of the dual-channel elevator scope in human studies should be considered.

## FUNDING

Amitabh Chak received research support from Olympus America, supported by NIH Grant DK02800.

**Conflict of interest:** Jeffrey Marks receives honoraria as a consultant for Olympus America.
